# Recent Progress in Bioprinting: From Bioink Design to Applications

**DOI:** 10.3390/bioengineering9120785

**Published:** 2022-12-09

**Authors:** Wujie Zhang

**Affiliations:** Biomolecular Engineering Program, Physics and Chemistry Department, Milwaukee School of Engineering, Milwaukee, WI 53202, USA; zhang@msoe.edu

Currently, there are more than 100,000 people on the US national transplant waiting list, and 17 people die each day waiting for an organ transplant [1]. Bioprinting offers an alternative approach to engineering tissues/organs for clinical applications. To define bioprinting, a working definition should be used, considering the bioprinting field is multidisciplinary and rapidly evolving. Three-dimensional bioprinting is an additive manufacturing technique that recapitulates the native architecture of tissues [2]. This editorial reviewed the recent progress in bioprinting based on a series of articles published in *Bioengineering* during 2020 and 2021.

A typical bioprinting process involves imaging, the design of bioprinted tissues, materials (for scaffolding) and cell selection, bioprinting, and the maturation of bioprinted tissues [3]. Bertolini et al. [4] reported an operative workflow from CT scans to 3D printed heart models. However, several challenges remain, such as segmentation, which can be time-consuming, and the accuracy and reproducibility of the whole workflow must be verified. 

An ideal bioink should exhibit the desired mechanical and rheological properties, good biocompatibility, and high bioprintability. Natural biomaterials are of great interest due to their biological origin, especially those that are components of a natural extracellular matrix. Benwood et al. [5] reviewed the properties of multiple types of natural biomaterial-based bioinks, including agarose, alginate, collagen/gelatin, chitosan, decellularized extracellular matrix (dECM), dextran, fibrin, gellan gum, hyaluronic acid, silk, and Matrigel. In particular, multi-component bioinks, combinations of different biomaterials designed to achieve better biological, rheological, and mechanical properties, were discussed. Somasekharan et al. [6] formulated and characterized a novel biomaterials-based multi-component bioink consisting of alginate dialdehyde, gelatin, and platelet-rich plasma. 

Among the above-mentioned natural biomaterials, collagen/gelatin, fibrin, and silk are protein/peptide-based. Proteins contain various functional groups which can be used for modification, physical gelation, and cross-linking. Stimuli-responsive and self-assembly protein-based bioink are two emerging classes of ink. For example, silk fibroin is able to form gel through the self-assembly of beta-sheets [7].

Cellulose, the most abundant biopolymer on earth, has also been explored for formulating bioink. For example, a novel cellulose-based bio-gel can now be used for bioprinting [8]. To produce the cellulose-based gel, cellulose was dissolved in an aqueous solution of NaOH and urea. This solution gels upon heating. Moreover, the addition of undissolved cellulose particles can be used a crosslinker to adjust the gel’s properties. Stenvall et al. [9] developed composite materials made of polypropylene (PP) reinforced with microfibrillated cellulose for the 3D printing of prostheses. Cellulosic nanomaterials, bacterial nanocellulose, cellulose nanofibers, and cellulose nanocrystals, have also drawn significant attention due to their biocompatibility, strong mechanical properties, high capacity for chemical modification, and so on [10]. Rosendahl et al. [11] developed a nanocellulose-based bioink using carboxylated cellulose nanofibrils. However, in vivo biodegradability remains a major challenge since the human body lacks the enzymes which can breakdown cellulosic materials [10].

Modifications of natural biomaterials have been investigated with the aim of improving their properties for bioprinting applications. For example, carboxylated agarose blended with a small amount of native agarose has been shown to exhibit ideal rheological behavior for bioprinting at physiological temperatures and notable gelling properties for printing complex free-standing objects with high stiffness [12].

Polyhydroxyalkanoates (PHAs), a class of polyester, have shown great potential for biomedical applications due to their biodegradability. PHAs have been used in drug delivery, vessel stenting, and tissue engineering. For example, a proximal femoral condyle scaffold has been 3D printed using Calcium phosphate/PHBV (a type of PHA) nanocomposites via selective laser sintering (SLS) [13]. 

Tissue vascularization is critical for clinical applications. Two major approaches are the induction of angiogenesis upon bioprinting and the direct printing of vascular channels [14]. Endothelial cells, such as human umbilical vein endothelial cells (HUVECs), are generally used to promote vascularization. However, the resolution and precise placement of different types of cells remain as challenges. 

It is difficult to engineer tubular-shaped tissues and organs. Bioprinting offers unique strategies to overcome the challenges faced by traditional approaches, such as casting and cell sheet technology. The bioprinting of tubular-shaped tissues/organs has been achieved through co-axial bioprinting, rod supporting bioprinting, and other bioprinting techniques [15].

When it comes to the applications of bioprinting in various fields, Nizioł et al [16], reported a novel bioink containing poly(N-isopropylacrylamide) (PNIPAAm) precursors, sodium alginate, and methylcellulose. The ink can be used to generate thermally responsive hydrogel scaffolds. Incorporated with an antimicrobial agent, this novel hydrogel scaffold shows great potential towards wound-healing applications. Additionally, bioprinting exhibits the potential for generating skin tissue-engineered products with the replicated dermal–epidermal junction (DEJ), considering DEJ plays various roles in skin homeostasis and function [17]. Significant progress has been made in cardiovascular [18], cartilage [19], musculoskeletal [20], and other tissue engineering fields in recent decades. Three-dimensional bioprinting has enabled the production of cardiovascular grafts, heart patches, values, and more [18]. Advancements have also been reported for engineering a full-thickness human cornea through bioprinting, as corneal transplantation is necessary for advanced stromal and endothelial disorders [21]. Overall, challenges related to biocompatible and bioprintability still remain. The complex regulatory pathway also remains a major challenge for the clinical translation of 3D-printed tissue/organ products [19,20]. 

It is worth mentioning that bioprinting has also been used for generating 3D tissue/organ models for other biomedical applications besides tissue engineering and regenerative medicine applications. For example, a bioprinted 3D cancer cell model has been developed for anti-cancer drug screening [11]. Interestingly, a 3D hydrogel-based in vitro tumor–stromal model has been produced, which recapitulates the angiogenic switch [22]. This model is specifically useful for studying the molecular mechanism of angiogenesis initiation.

Moving forward, significant progress in novel bioink design and bioprinting methods are required to produce biocompatible and fully vascularized tissues/organs. In particular, the precisely controlled placement of various types of cells within the scaffold to fully replicate a native tissue/organ needs further investigation. The application of artificial intelligence (AI) and machine learning (ML) shows great potential in improving the bioprinting process design [19]. Lastly, 4D bioprinting, with a further dimension of transformation over time (such as functionalities changing over time under external stimuli), is expected to be the next generation of technology [18,23].
